# Rare causes of anisocoria: Ipratropium bromide and Angel’s trumpet

**DOI:** 10.14744/nci.2020.26428

**Published:** 2021-11-15

**Authors:** Arzu Ekici, Busra Caglar, Ozlem Kara, Arzu Oto, Nevin Kilic

**Affiliations:** 1. Department of Pediatric Neurology, University of Health Sciences, Bursa Yuksek Ihtisas Training and Research Hospital, Bursa, Turkey; 2. Department of Pediatrics, University of Health Sciences, Bursa Yuksek Ihtisas Training and Research Hospital, Bursa, Turkey; 3. Department of Pediatric Endocrinology, University of Health Sciences, Bursa Yuksek Ihtisas Training and Research Hospital, Bursa, Turkey; 4. Department of Pediatric Critical Care, University of Health Sciences, Bursa Yuksek Ihtisas Training and Research Hospital, Bursa, Turkey

**Keywords:** Angel’s trumpet, anisocoria, ipratropium bromide

## Abstract

It is considered a neurological emergency when a patient presents with anisocoria. It is important that the anisocoria whether or not accompanied by the neurological findings. Other reasons of anisocoria should be considered when the absence of neurological or ophthalmological signs such as change of mental status, hemiparesis, ophthalmoplegia, ptosis. Herein we report two cases of temporary anisocoria due to inhaler ipratropium bromide and Angel’s trumpet.

**A**nisocoria is defined as an inequality in the size of the pupils anisocoria has a wide range of causes from benign to immediately life-threatening. Cerebrovascular accident, oculomotor nerve palsy, Adie tonic pupil, previous trauma/eye surgery, cerebral aneurysm, multiple sclerosis, topical/systemic drug-induced mydriasis, or exposure to plant toxins can cause unilateral mydriasis [1]. Herein we report two cases of temporary anisocoria due to inhaler ipratropium bromide and Angel’s trumpet.

**Case 1** – A healthy 17-year-old boy was admitted to the emergency department complaining of sudden onset of blurry vision and eye burning in the left eye. He had left unilateral mydriasis without any other neurological deficits (Fig. 1A). There was no history of trauma or medication use in this patient. Extraocular muscles were intact. Funduscopic examination and visual acuity were normal. Cranial and diffusion-weighted magnetic resonance imaging were initially normal. When the patient was questioned again, it was learned that his complaints arose when he was mowing Angel’s trumpet, locally known as white datura (Fig. 2). He said that he did not take it orally. The patient’s mydriasis improved within 24 hours after initiation of symptoms, and he no longer complained of blurry vision (Fig. 1B).

**Figure 1.**
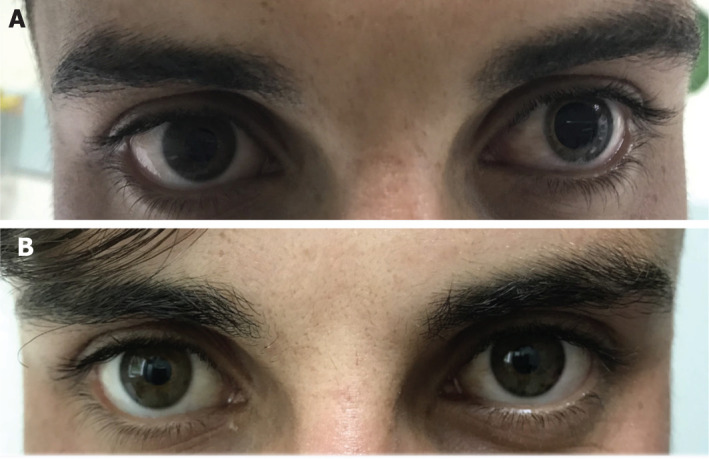
**(A)** Left unilateral mydriasis induced by Angel’s trumpet. **(B)** Anisocoria was completely resolved.

**Figure 2.**
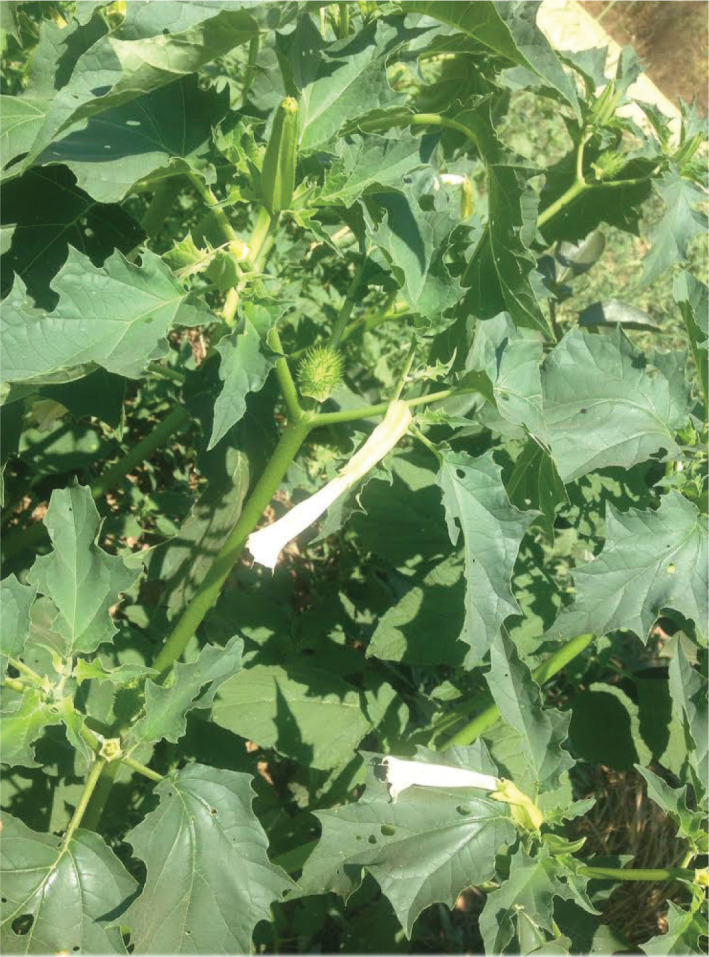
Angel’s trumpet.

**Case 2** – A 5-month-old girl was presented to the emergency department complaining of cough and wheezing. Physical examination revealed tachypnea, dyspnea, expiratory wheezes, and fine diffuse crackles on lung auscultation. She was taken to intensive care unit. There was infiltration in the left lung on chest x-ray. Intravenous ampicillin-sulbactam and nebulized salbutamol- ipratropium bromide with a face mask was initiated. She was noted to have left unilateral mydriasis on the second day of hospitalization (Fig. 3A). She had no other neurological deficits. Eye movements and optic disc were normal. Cranial computed tomography revealed no abnormalities. Ipratropium bromide treatment was discontinued. Her anisocoria was completely resolved twelve hours after discontinuing the ipratropium bromide (Fig. 3B).

**Figure 3.**
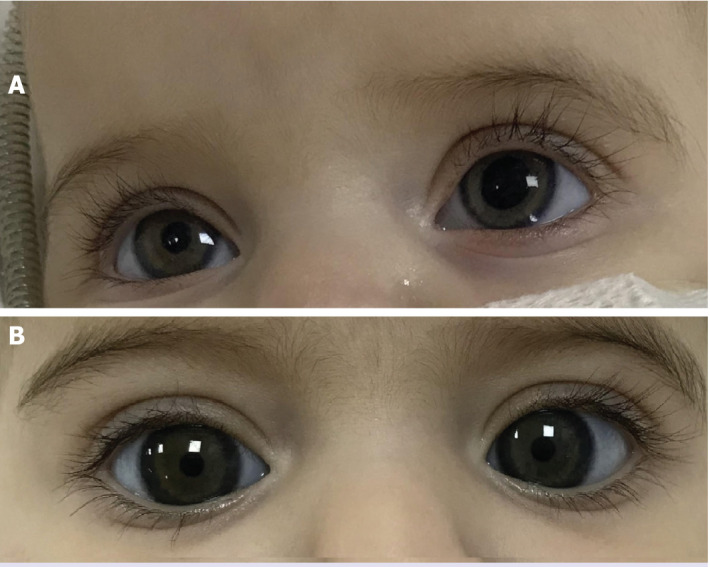
**(A)** Left unilateral mydriasis induced by ipratropium bromide **(B)** Anisocoria was completely resolved after discontinuing ipratropium bromide.

## Discussion

Trauma, local and systemic disorders can cause anisocoria. Systemic causes of anisocoria are neurological or vascular disorders, usually associated with raised intracranial pressure. Therefore it is considered a neurological emergency when a patient presents with anisocoria. The clinician primarily wants to exclude intracranial pathology such as intracranial aneurysms, hemorrhage, demyelinating diseases or brain tumors [1]. It is critical to note whether or not the anisocoria is accompanied by neurological abnormalities. Lokal causes such as afferent pupillary defects, glaucoma should be consider in anisocoria with visual symptoms. They can be excluded based on detailed ophthalmoscopic examination [2]. Trauma and drug use must be questioned. Pharmacological agents such as pilocarpine, cocaine, tropicamide, dextromethorphan, and ergolines are one of the benign etiologies of anisocoria [3].

Plants of the genera Brugmansia and Datura may also induce anisocoria. All parts of the Brugmansia genus, often known as angel’s trumpets, are poisonous. It contains high concentration of the alkaloids atropine, scopolamine, and hyoscyamine. Somatic symptoms (tachycardia, mydriasis, hypertonia, respiratory disturbances, and vomiting), pyramidal signs, hallucinations, encephalopathy, convulsion, and death have been reported due to Angel’s trumpet [4, 5]. In our case 1, it was learned that his complaints arose when he was mowing the angel trumpets. He said that he did not take it orally. Systemic and local intoxications of Angel’s trumpet have been described. However, no previous reports of anisocori connected with inhaler angel’s trumpet have been found. At the same time, intoxication from smoking jimson Datura stramonium has been documented [6]. In our case 1, anisocoria may be due to inhalation alkaloids released during mowing, or the patient may be hiding that he received orally. Because these alkaloid plants have been used as hallucinogen [5].

Etiology of anisocoria is ipratropium bromide in our case 2. A computed tomographic scan of her head was normal. After the patient stopped using ipratropium, the anisocoria disappeared within 12 hours. Ipratropium bromide is a derivative of atropine and competitive antagonists of acetylcholine. Dizziness, fatigue, headache, and nervousness are the nervous system side effects. When the mask is not adjusted properly, nebulized ipratropium bromide might cause mydriasis secondary to contact with the eye. When the conjunctiva is exposed to ipratropium, mydriasis and acute glaucoma can occur [7, 8].

It is often considered that sudden onset anisocoria is a sign of uncal herniation or intracranial pathology. As a result, the physician requests urgent neuroimaging. We performed neuroimaging in both of our patients to exclude the intracranial pathology, although there was no other neurological finding. A thorough neurological and ophthalmological examination and detailed history may avoid unnecessary imaging studies and therapeutic intervention. When there are no neurological or ophthalmological symptoms, such as change of mental state, hemiparesis, ophthalmoplegia, or ptosis, other causes of anisocoria should be examined. The benign etiologies will also be supported by transitory anisocoria.
